# Causal Context Presented in Subsequent Event Modifies the Perceived Timing of Cause and Effect

**DOI:** 10.3389/fpsyg.2017.00314

**Published:** 2017-03-07

**Authors:** Hiroyuki Umemura

**Affiliations:** Human Informatics Research Institute, National Institute of Advanced Industrial Science and TechnologyTsukuba, Japan

**Keywords:** causality, time perception, temporal order judgements, vision, causal perception

## Abstract

The effect of perceived causality on other aspects of perception, such as temporal or spatial perception, has interested many researchers. Previous studies have shown that the perceived timing of two events is modulated when the events are intentionally produced or the causal link between the two events was known in advance. However, little research has directly supported the idea that causality alone can modulate the perceived timing of two events without having knowledge about causal links in advance. In this study, I used novel causal displays in which various types of causal contexts could be presented in subsequent events (movement or color change of objects). In these displays, the preceding events were the same (ball falling from above), so observers could not predict which subsequent events displayed. The results showed that the perceived causal context modulated the temporal relationship of two serial events so as to be consistent with the causal order implied by the subsequent event; ball hit the floor, then objects moved. These modulations were smaller when the movements implied preceding effect of the falling ball (e.g., wind pressure). These results are well-suited to the Bayesian framework in which the perceived timing of events is reconstructed through the observers' prior experiences, and suggest that multiple prior experiences would competitively contribute to the estimation of the timing of events.

## Introduction

When one sees a ball flying toward a glass and breaking it, one also perceives causality between these two events, namely the ball hit and broke the glass. The perception of causality has been attracted many researchers since Michotte showed that simple visual stimuli could give the impression of a causal connection between events (Michotte, 1963; Wagemans et al., 2006). It is relatively recent that studies about the effects of causal perception on other perceptual process such as temporal (Eagleman and Holcombe, 2002; Haggard et al., 2002; Buehner, 2012; Bechlivanidis and Lagnado, 2013) and/or spatial perception (Scholl and Nakayama, 2004; Buehner and Humphreys, 2010) have appeared. However, such studies have obtained various interesting findings. One type of experimental evidence for the effect of causality on temporal perception was introduced by Haggard (Haggard et al., 2002), who showed that the perceived timing of two causally linked events converged together such that participants perceived voluntary movements (key-press) as occurring later and their sensory consequences (tone) as occurring earlier than they actually did. Although they offered an explanation in which the binding effect is rooted in the motor system and is driven by the intentional action planning for this “intentional binding” phenomenon (Haggard et al., 2002; Haggard, 2005; Engbert and Wohlschläger, 2007; Moore and Haggard, 2008), several researchers proposed that causality should be the root of this temporal attraction, and that the intentional binding should be a subset of a more general “causal” binding (Eagleman and Holcombe, 2002; Buehner, 2012). Buehner (2012) demonstrated that both a self-causal condition, in which participants themselves pressed a button, and a machine-causal condition, in which a machine pressed a button instead of a participant, produced more temporal binding than a non-causal condition did. This indicates that temporal binding occurs without one's intention. Further, he discussed that the prediction of future events that is driven by the causal knowledge should contribute to these results.

If causality, however, is the root for attracting the timing of two events, it can also be predicted that temporal attraction should be observed even when the prediction is not available. The notion of causal binding has its basis on Bayesian inference, which enables us to model the uncertainty about the world by combining prior probabilities with sensory evidence to infer the most probable interpretation of the environment (Knill and Richards, 1996). The brain uses prior knowledge that events known to be causally related are more likely to occur closer in time than unrelated events are to resolve the ambiguity in time perception. This shifts the estimates of “cause” and “effect” toward one another. The Bayesian model has explained a variety of phenomena in vision (Yuille and Bülthoff, 1996; Weiss et al., 2002; Welchman et al., 2008), sensory motor control (Körding and Wolpert, 2004, 2006), multimodal integration (Ernst and Banks, 2002; Ernst and Bülthoff, 2004) and temporal perception (Miyazaki et al., 2005). In the Bayesian framework, prediction of the future that is driven by causal knowledge can be formulated as the selection of a prior probability according to a certain context. However, in this framework, an appropriate prior probability can be selected even after an observation. This means that we can expect the situation in which temporal modulation is observed for stimuli in which the causal context is not given in advance.

Several previous studies on causality perception have employed Michotte's launching display in which one object moves toward another stationary object until they are adjacent, then the moving object stops and the stationary one starts moving along the same path, and in a typical case, a we perceive collision in the display (Michotte, 1963; Scholl and Nakayama, 2002; Choi and Scholl, 2006). Some researchers proposed more informative causal displays such as “pulling” or “enforce disintegration” (White and Milne, 1997, 1999; Scholl and Tremoulet, 2000). To my knowledge, a recent study by Bechlivanidis and Lagnado (2016) is the only one which showed that causal contexts could modulate the temporal relationship of two events without advanced information. They too used Michotte-like stimuli. Their stimuli had an additional object between the two objects. Specifically, the leftmost object moved toward the middle one, with the collision of the leftmost object with the middle object, the rightmost object started moving, and the middle object started moving after a 350 ms delay. Participants perceived the start of the middle object before the start of rightmost object despite the delay, indicating that the perceived order was reordered based on the causal order.

The stimuli in the present study were devised based on these displays. However, there were several differences between previous stimuli and the present stimuli. One of notable differences is that various and informative causal contexts were represented by objects' movements or feature changes in subsequent events. I thought that stimulus change in subsequent event might be able to retrospectively imply a context of causal links. For example, if participants perceive that objects were moved as if they were blown by the wind pressure of a preceding object, this suggests that they would perceive that objects started moving before the contact of the preceding object. For the purpose, I created several types of movement which would give different interpretations. Therefore, the information in the stimuli was increased by simulating a scene in a 3-dimensional view and by simulating the motion by multiple objects. Using these stimuli, I demonstrated that these different contexts have different effects on temporal perception, even though this did not allow observers to predict the causal context in advance. Moreover, in the experiments by Bechlivanidis and Lagnado (2016), each participant experienced the causal display only a few times, such that it was unclear whether the causal context is effective even after the participants repeatedly observed the causal displays. In the present study, participants repeatedly observed causal displays, which would reveal whether the effect of causal contexts on the temporal perception were rigid or not, and it made it possible to quantitatively estimate shifts of perceived timings of the contact.

## Materials and methods

**Figure 1:**
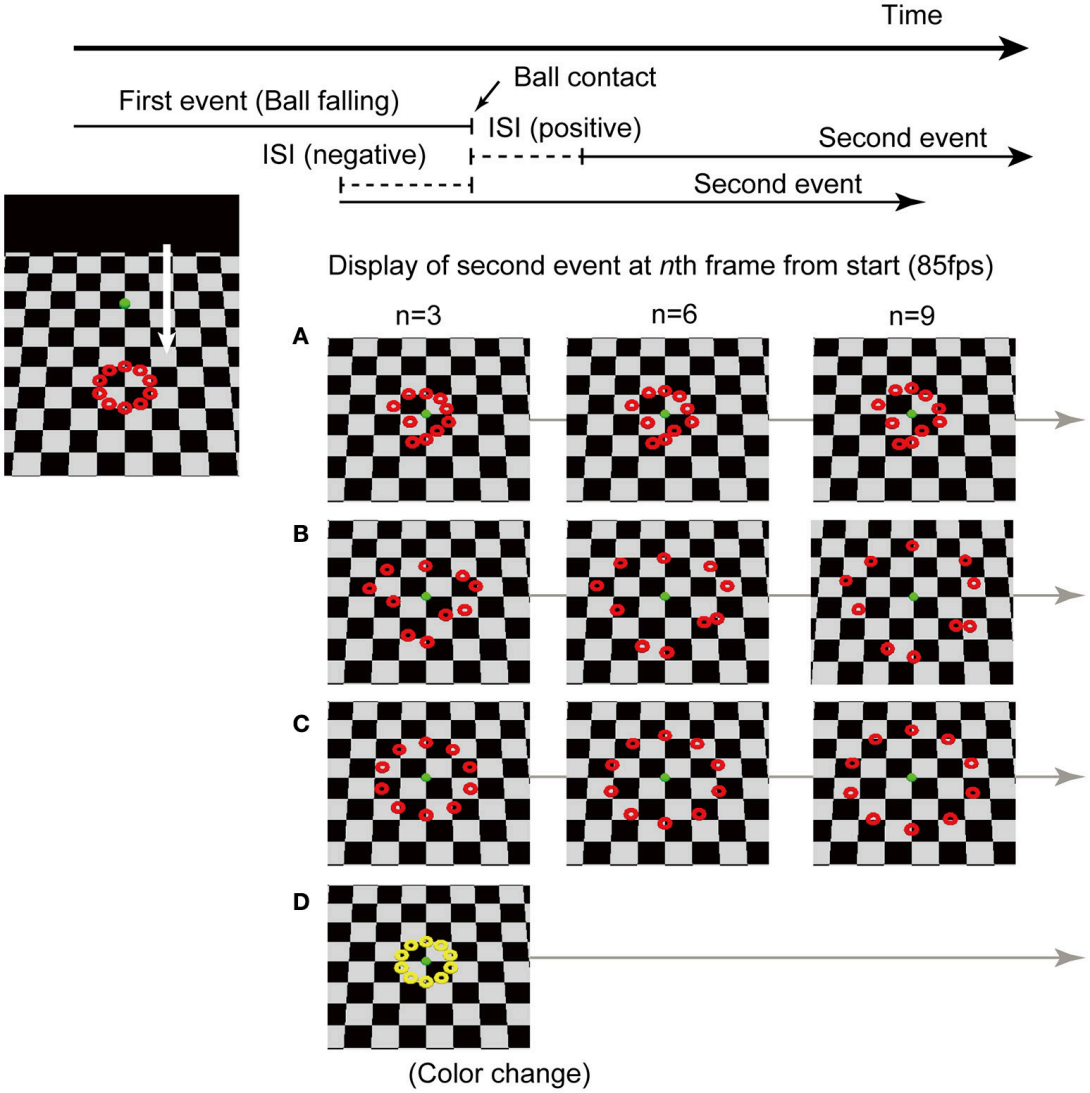
**Stimulus design and procedure of the temporal order judgement task**. The preceding event was a ball falling from above for all types of stimuli. The objects (rings) positioned on a floor started to move or change their color around the time when the falling ball contacted the floor. Four types of stimuli were prepared. **(A)** Rings started short rapid movements in random directions for a short time; **(B)** rings rapidly moved toward the outer edge in random directions and distances; **(C)** rings moved with a straight and smooth motion toward the outer edge; and **(D)** rings changed in color. The two events occurred serially with controlled inter-stimulus intervals (ISI). A negative ISI indicates that the second event started before the ball contacted the floor.

In this study, using computer generated animation, the falling of a ball was utilized as the preceding event. In subsequent events, 10 rings on the floor moved or changed their color. I prepared four types of displays (see Figure 1, and Videos S1–S8 in the Supplementary file). For type A, B, and C stimuli, the preceding event was a ball falling from above and the subsequent event was the movement of 10 rings on the floor. Three types of ring movements could be interpreted as different causal contexts. For type D stimuli, a ball fell and the rings changed their color. The timing of the end of the first event to the start of the second event was chosen from nine inter-stimulus interval (ISI) conditions. Participants were required to judge whether the end of the first event or the start of the second event occurred first (temporal order judgement task).

### Participants

Twenty-seven observers (aged 20–37 years), naïve to the experimental purpose, participated in the present study. All the experimental procedures were approved by the Ethics Committee for Human and Animal Research of the National Institute of Advanced Industrial Science and Technology (AIST). All subjects provided written informed consent in accordance with the Declaration of Helsinki before the experiment. Based on prior literature and my other works, it was decided ahead of time to select a minimum of 25 participants for the experiment. Thirty participants were recruited and three participants canceled. All data collection was completed before commencing the statistical analyses.

### Apparatus and setup

The experiments were conducted in a dimly lit room. A Windows PC was used to control the stimulus presentation on a CRT monitor (Sony 24″ GDMFW900) placed 40 cm from the observer. The height of the center of the display and the eye-height of each participant was adjusted using a chin-rest. The CRT's refresh rate was 85 Hz and resolution was 1,024 × 768. 10 keys were used for making responses.

### Stimuli and tasks

The visual stimuli were sequences of animation which consisted of two serial events: The fall of a ball from above and the movements of 10 objects placed on the floor. The scene in the animation was three-dimensionally rendered and the viewpoint was placed at 45° elevated from the horizontal floor. The falling ball was a shaded green sphere subtending 1° in diameter. The 10 objects were shaded red rings, each subtending 1° in diameter. The 10 rings were placed along a circle whose width was 4°. The preceding event was the same for all the stimuli; the ball fell from 12° above from the center of the rings (= fixation) and stopped when the ball touched the floor at the center of the rings. The ball traveled this distance in 142 ms. Four types of subsequent events were prepared. For the Type A stimulus (Videos 1, 2), the rings started short rapid movements for random distances (about −1 to 1° in visual angle) for a short time (about 35 ms) and then the magnitude of the movements rapidly decreased and the rings continued to tremble. For the Type B stimulus (Videos 3, 4), the rings started rapid movement toward the outer edge with slight randomness in directions. They rapidly moved at a visual angle of about 5°, nearly 70 ms later, and then the magnitude of the movements rapidly decreased and the rings continued to tremble. In Type C (Videos 5, 6), the rings moved with a straight and smooth motion toward the outer edge. They moved by about 5° in visual angle, nearly 70 ms later, and then continued spreading. For the Type D stimulus (Videos 7, 8), the rings changed their color from red to yellow. In each trial, the static rings on the ground were initially displayed. Then, the ball fell down and the rings started moving or changed color, with various delays. The movement was generated in each of trials. The ISIs were chosen from −118, −94, −71, −47, −24 0, 24, 47, and 71 ms, where negative ISIs indicate that a subsequent event started before the ball contacted the floor. In the supplementary files, Videos 1, 3, 5, and 7 are picked up from stimuli with ISI = 0 ms, and Videos 2, 4, 6, and 8 are ISI = −71 ms. The image quality was slightly degraded in these supplementary videos as compared to those in the original displays because of video conversion, and their temporal resolution was lessened from 85 to 60 fps.

### Procedure

#### Temporal order judgement task

Participants judged the temporal order of the ball contacting the floor (stopping) and the start of the rings' movements using a two-alternative forced-choice design. Participants pressed the “4” key when they perceived the rings starting movements before the ball contacted the floor, or pressed the “6” key when they perceived the rings starting movements after the ball contacted the floor. Participants conducted several practice trials which included all of the four types of events. In the experiment, the four types of stimuli with nine ISIs were displayed in random order. Participants were shown all combinations of stimuli type and ISIs (4 × 9) nine times in random order, such that they conducted 324 trials. These trials were divided into three blocks. No feedback was provided during the task.

#### Causal judgement task

The causal judgement task was conducted after all the temporal order judgement tasks were completed. In this task and in the subsequent interview, animations with an ISI of −118, 0, or 71 ms were displayed for participants. Participants choose a higher score (max 9) when they strongly perceived causality (instruction: “If you experience that the falling ball caused the rings' movement or color change, please give a high rating. However, if you feel there was no causality between the movement or the color change of the rings and the falling ball, please give the lowest score of 0. You can choose one of ten scores for your judgements. If you experience a slight strangeness, please decrease the scores in a descending order from 9. If you do not experience causality but think that a less causal stimulus would exist, please increase the score in an ascending order from 0. You need not be concerned how the falling ball caused the movement or color change. I will ask for your interpretation about perceived causality later. As far as possible, you should recall the perceived causalities during the temporal order judgement tasks conducted before and rate the causality based on them.”). All the animations were rated twice or once (for the first five participants), in random order, such that the number of trials were 18 or 9.

#### Interview

An interview was conducted after the causal judgement task. The same animations that were used in the causal judgement task (ISI of −118, 0, or 71 ms) were used for the interview. An experimenter sat next to a participant, showed the participant one of the animations, and asked him/her to express how the falling ball caused the second event when they perceived causality. Participants could freely express the causality they experienced. When the participants did not experience causality, they could simply report that they did not experience causality. After the participant expressed their perceptions of causality for all the animations, the experimenter asked the participants to confirm the following point if they did not report that the rings were moved by wind pressure or repulsing forces from the falling ball in the ISI = −118 ms sessions of Type A, B, or C stimuli: “Didn't you perceive such a force or pressure during the temporal order judgement tasks?” The order of presentation about the stimulus type was randomized but the order of ISIs was ascending order from ISI = −118. Participants were instructed about which type of animations would be shown to them next, and could ask the experimenter to repeatedly show a stimulus.

## Results

### Temporal order judgement task

**Figure 2:**
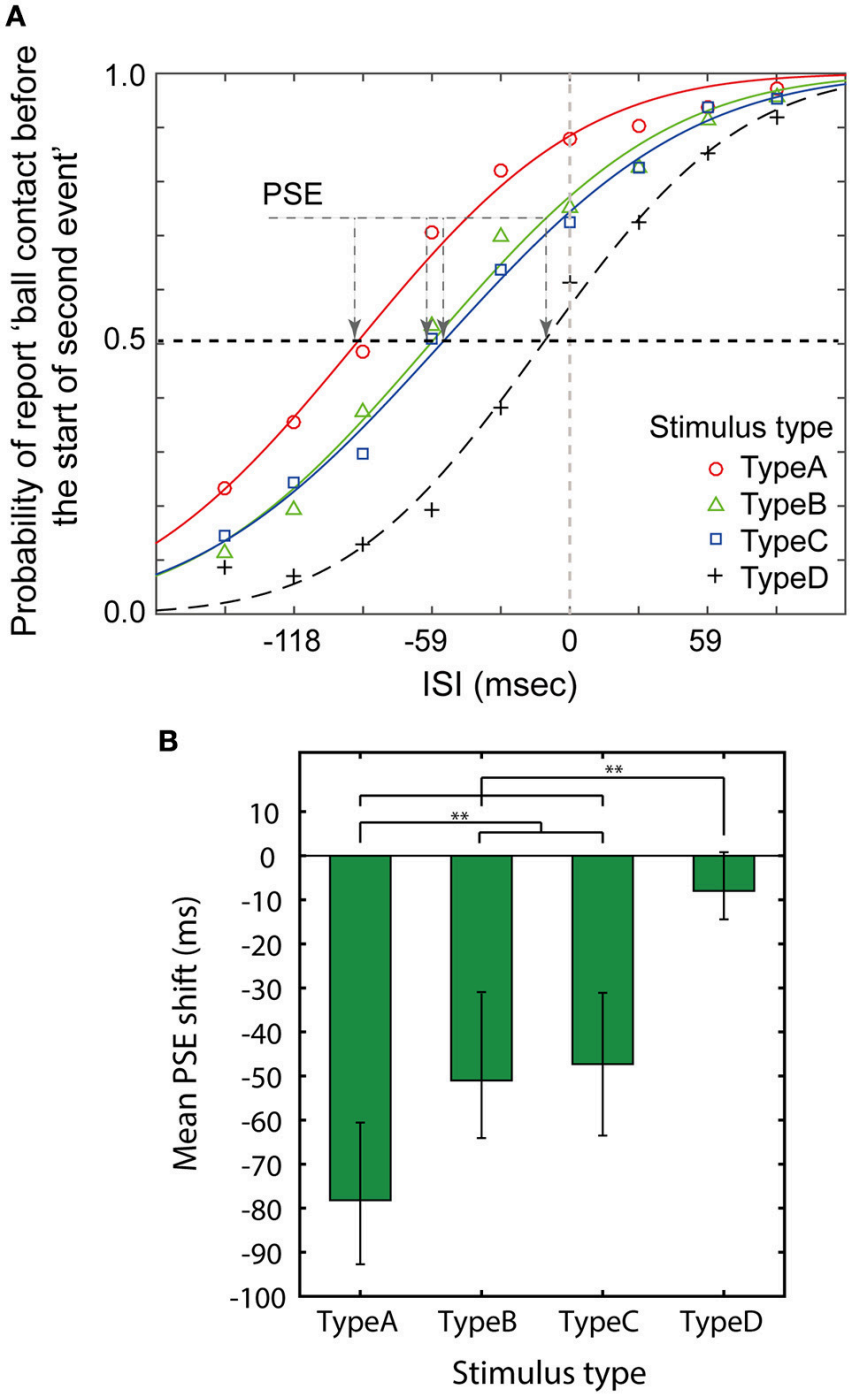
**Results of the temporal order judgements task. (A)** The average proportion of trials in which participants reported that the end of the first event (ball contacting) was earlier than the start of the second event is plotted against the ISI. The curves in this figure are fit for the mean proportions over all the participants. **(B)** Averaged PSE shift from zero for all participants. Error bars indicate 95% CI. ^**^Indicates *p* < 0.01.

Figure 2 shows results of the temporal order judgement experiment in which participants were required to answer whether the rings started moving before the falling ball contacted the surface, or the rings started moving after the ball made contact. The proportions of trials in which the end of the preceding event was earlier than the start of the subsequent event were plotted and the point of subjective equality (PSE), which indicates that the end of the first event and the start of the second event were simultaneous, was calculated by fitting with a cumulative Gaussian function as an index of the extent to which the timing of the end of the first event and the start of the second event converged (Figure 2A). PSEs were calculated for each participant. Their averages and *SD*s for each stimulus type were as follows: A: −78.8 (*SD* = 41.2), B: −48.7 (*SD* = 37.6), C: −45.3 (*SD* = 34.0), and D: −9.5 (*SD* = 22.4). They are presented in Figure 2B. The PSEs were analyzed with repeated measures ANOVA with one factor (stimulus type). The repeated measures ANOVA revealed a significant main effect of stimulus type [*F*_(3, 78)_ = 25.414, *p* <.001, η^2^ = 0.494]. A *post-hoc* comparison with a Bonferroni adjustment revealed a significant difference between the D and A, B, or C stimuli (*p* < 0.001, for all pairs), and revealed significant differences between A and either B or C (*p* = 0.007, *p* = 0.001, respectively), but not between B and C (*p* > 0.5).

### Causality rating task and interviews

Table 1 shows the summary of these causality ratings. Although results for the 71 ms condition were displayed in Table 1, they were not analyzed here, because they should have little contribution to the present study. One-way ANOVA for rated causality with ISI = 0 ms showed a significant effect of stimulus type [*F*_(3, 78)_ = 17.049, *p* < 0.001, η^2^ = 0.396], and a *post-hoc* comparison with a Bonferroni adjustment revealed significant differences between D and A, B, or C stimuli (*p* = 0.007, *p* = 0.001, *p* < 0.001, respectively), and between A and C (*p* = 0.004). The ANOVA for the rated causality for ISI = −118 ms among stimulus types A, B, or C showed a significant effect of stimulus-type [*F*_(3, 78)_ = 7.906, *p* < 0.001, η^2^ = 0.233], and a *post-hoc* comparison with a Bonferroni adjustment revealed significant differences between A and B (*p* = 0.049), A and C (*p* = 0.026), B and D (*p* = 0.005), and C and D (*p* = 0.008), but not between B and C (*p* > 0.5), and A and D (*p* > 0.5).

**Table 1:** **Mean rated causalities in the causality rating task**.

**Stimulus type**	**M (SD)**	**95% CI**
**ISI = 71**		
Type A	5.4 (2.2)	[4.4, 6.2]
Type B	6.0 (2.0)	[5.2, 6.8]
Type C	6.0 (2.5)	[5.0, 7.0]
Type D	5.1 (2.2)	[4.2, 5.9]
**ISI = 0**		
Type A	7.1 (1.7)	[6.4, 7.8]
Type B	7.4 (1.7)	[6.7, 8.1]
Type C	8.1 (1.2)	[7.5, 8.6]
Type D	5.0 (2.3)	[4.1, 6.0]
**ISI = −118**		
Type A	3.3 (2.5)	[2.3, 4.2]
Type B	4.6 (2.9)	[3.4, 5.8]
Type C	4.5 (2.3)	[3.6, 5.4]
Type D	2.6 (2.5)	[1.6, 3.6]

The interpretations of the participants were categorized into the expressions shown in Table 2. Results for 71 ms were not displayed because of space limitations. No participants reported multiple interpretations for a stimulus. For Type A, B, and C stimuli in the ISI = 0 ms condition, most participants answered that it looked as if the rings were moved by the impact of the ball contacting the surface; some perceived animacy, such as the rings running away from the ball. For Type D stimuli, most participants perceived that the ball tripped a switch on the floor to change the color of the rings, but the rated causality was relatively small compared to that for type A, B, and C stimuli. Some participants reported the effect of the falling ball before the ball contacted the surface (ISI = −118 ms) for Type B and C with these interpretations; it looked as if the rings were blown by the wind pressure caused by the falling ball, or the rings were burst by magnetic repulsion. Similar answers were obtained for Type A stimuli but the number was small. The numbers of participants who mentioned these preceding and subsequent effects are shown in Table 2. Although there were a small number of unpredicted observations, these answers were generally consistent with the intention of the experimenter in creating these movements.

**Table 2:** **Reported interpretations and numbers of participants who stated the interpretations**.

**Stimulus type**	**Interpretation**	***n***	**Total**
**ISI = 0**			
Type A	Trembled by collision impact	21	
	Run away from or avoid a ball	3	
	Others	2	
			26
Type B	Scattered by collision impact	21	
	Run away from or avoid a ball	4	
	Other	1	
			26
Type C	Spread by collision impact	26	
	Other	1	
			27
Type D	Floor is the switch	22	
	Others	2	
			24
**ISI = −181**			
Type A	Run away from or avoid a ball	5	
	Wind pressure	3	
	Magnetic repulsion	2	
			10
Type B	Run away from or avoid a ball	8	
	Wind pressure	3	
	Magnetic repulsion	3	
	Other	1	
			15
Type C	Wind pressure	5	
	Magnetic repulsion	2	
	Run away from or avoid a ball	2	
	Experienced causality but could not express	3	
			12
Type D	There is a (transparent) switch above the floor	3	
			3

### Discussion

These analyses revealed negative shifts of PSE in Type A, B, and C stimuli as compared to that in Type D. This indicates that participants' perceived temporal orders changed according to the causal order; a ball hit the ground and then rings moved. Additionally, the results of the analysis showed interesting results between the PSEs of A and B or C; the shift in the PSE in Type A was significantly smaller than that in others. These three types of stimuli had similar rated causality and received similar interpretations when ISI = 0 ms (i.e., no delay). These differences are thought to be caused by contexts from a falling ball before contacting the surface, which were described as “wind pressure” or “magnetic repulsion.” These were more frequently reported for Type B and C stimuli than for Type A stimuli when ISI = −181 ms. Here, we can hypothesize that these preceding effects would conflict with the effect of causality occurring at the time of impact of the ball. Thus, this preceding effect would weaken the effect of subsequent causality, because these preceding effects would be able to move the rings before the ball contacted the surface.

**Figure 3:**
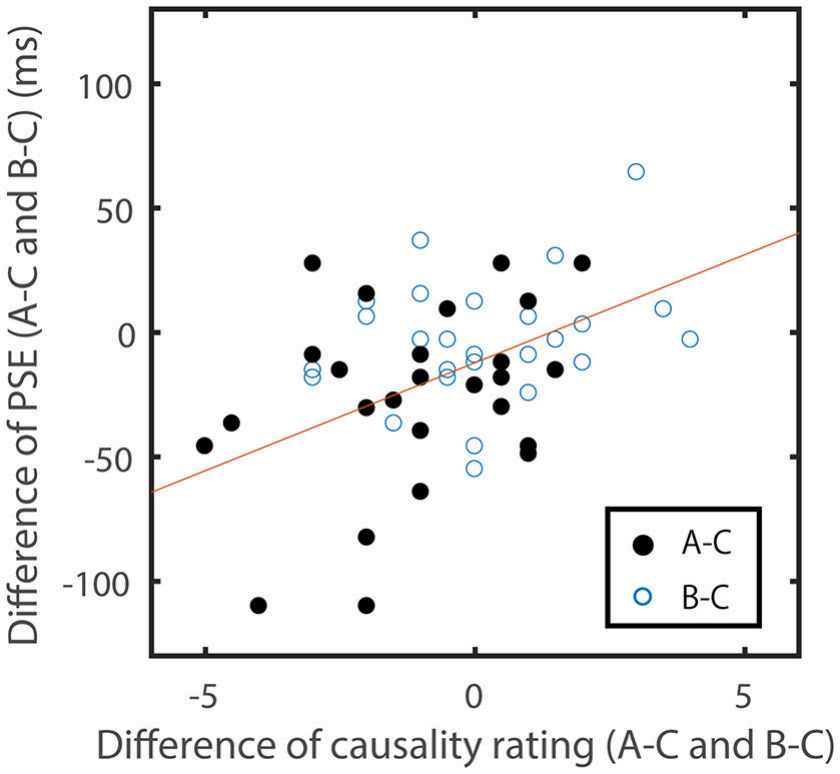
**Correlation scatter plot for causality ratings for ISI = −181 and shifts of PSE in each participant**. Differences between the causality ratings between A or B and C (A–C and B–C) are plotted against the differences between the shifts of PSE in A or B and C (A–C and B–C). The Pearson correlation coefficient between the two variables was 0.471 (*p* <0.01).

One type of evidence for this hypothesis was the significantly smaller rated causality for Type A stimuli in the preceding condition (ISI = −118 ms). Here, it is also expected that there would be a correlation between these causality rating for ISI = −181 ms and the shift of PSE in each participant. To examine this, I used the rating and PSE of Type C as a baseline, and calculated the difference between the baseline and those of Type A and B for each participant (Figure 3). These differences in the PSE and ratings showed a significant correlation (*n* = 54, *r* = 0.47, *p* < 0.001), which also suggests that the weaker perceived preceding causality in Type A might shift the PSE toward a negative value. However, when the ratings and PSEs for Type D were used as a baseline, the correlation was relatively small (*n* = 81, *r* = 0.28, *p* = 0.01). This might be because the difference of the criteria used to evaluate causality for Type D and for other three types varied more largely among participants. We should therefore be cautious while comparing causality ratings among different situations.

I conducted another comparison to confirm this hypothesis. I created a subgroup which consisted of 10 participants who did not report the preceding effect of the falling ball for any type of stimuli. If the preceding causality affected the shift of the PSE, it would be expected that this subgroup would show smaller or no differences between Type A and B or C stimuli. Although this comparison is not sufficient evidence for the difference between A and B or C, it would be necessary to examine whether the difference arose from a preceding effect of causality. In the subgrouping, 10 participants who did not report the preceding effect of the falling ball for any type of stimuli even after the interpretation was suggested by the experimenter were chosen. They were compared with the rest of the participants (*n* = 15). Two participants, who were aware of the interpretation after the experimenter's suggestion were removed from both groups.

**Figure 4:**
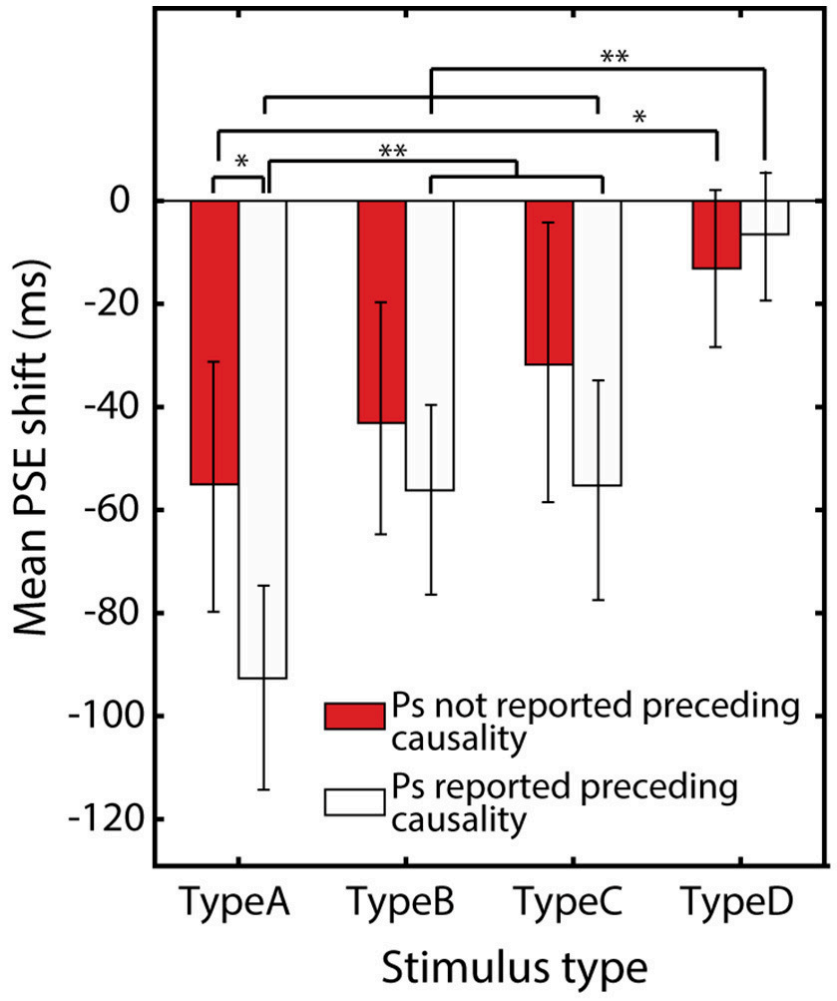
**Results of the temporal order judgement with subgrouping**. Averaged PSEs for the subgroup which consisted of participants who were not aware of the preceding effect of the falling ball (*n* = 10) and for the rest of the participants (*n* = 15). Two participants who were aware of the interpretation after the experimenter's suggestion were removed from both the groups. Error bars indicate 95% CI. ^**^Indicates *p* < 0.01 and ^*^indicates *p* < 0.05.

The averaged PSE shift for each subgroup are shown in Figure 4. Their averages and *SD*s for each stimulus type in the participants who reported the preceding causality were A: −55.3 (*SD* = 41.2), B: −43.5 (*SD* = 29.6), C: −31.7 (*SD* = 27.5), and D: −12.9 (*SD* = 25.3), and averages and *SD*s for the rest were A: −95.3 (*SD* = 41.9), B: −58.8 (*SD* = 39.0), C: −57.6 (*SD* = 46.0), and D: −7.0 (*SD* = 25.1). A repeated measures ANOVA with one-within and one-between factors (stimulus-type and group) revealed the significant effect of the stimulus type [*F*_(3, 69)_ = 21.649, *p* < 0.001, η^2^ = 0.485], the group [*F*_(1, 23)_ = 74.689, *p* < 0.001, η^2^ = 0.765], and their significant interaction [*F*_(3, 69)_ = 2.829, *p* = 0.045, η^2^ = 0.11]. *Post-hoc* comparisons with a Bonferroni adjustment revealed no significant differences between each pair of A, B, and C (all *p* > 0.5) in the subgroup of participants who did not report causality. There was a significant difference between A and D in the subgroup (*p* > 0.05). In contrast, the results of the comparisons for the rest of the participants were similar to those in the comparison for all participants (see Figure 4 for these results). Comparisons of the PSE for each type of stimulus between the subgroup of participants who did not report causality and the rest showed a significant difference only in the Type A stimulus (*p* = 0.018). These results supported the hypothesis that preceding effects would conflict with the effect of causality occurring at the time of impact of the ball. It should be noted that, if the preceding effect alone was decreased in this subgroup, we should expect a larger effect of subsequent causal contexts in the subgroup; i.e., the PSEs of Type B and C would shift toward a negative value. However, the PSEs of Type A largely shifted toward a positive value, and the PSE of Type B and C also shifted toward positive value in this subgroup. I speculate that this should indicate that the effects of subsequent events were also weak for these participants who were not report the preceding causality, and that they were generally unsusceptible to the effects of causality. The causality ratings were as follows: A: 2.8, B: 4.15, C: 3.25, and D: 1.45, for each type of stimulus in the subgroup of participants who were not aware the preceding causality, and A: 3.9, B: 5.9, C: 5.33, and D: 3.9 for the rest of the participants. These relatively low mean causality rating scores even for Type B and C stimuli in the subgroup might indicate that participants who did not report the preceding causality were generally unsusceptible to the effects of causality.

## General discussion

The present study showed that the perception of causality leads to the reconstruction of the temporal order of serial events consistent with the causal context; that is, a cause precedes its effect. These results are generally accorded the report by Bechlivanidis and Lagnado (2016). Previous studies (Eagleman and Holcombe, 2002; Shimojo, 2014) suggested that the brain should reorganize the event sequence in accordance with a contextual or causal framework if two events have sufficient causal links. In the present experiment, the stimuli representing the causality by object movement could offer sufficient causal links without the contribution of prediction or intention. On the other hand, the stimulus with color change could not modulate the temporal relationship, even though most of participants could state the interpretation about the perceived causal link.

Because the causal contexts were given in subsequent events, their effect should be formed by integrating information presented within a short temporal window, i.e., retrospectively. Such retrospective effects relating causality perception have been reported in several studies (Choi and Scholl, 2006; Buehner and Humphreys, 2010; Kim et al., 2013). For example, Choi and Scholl (2006) demonstrated that the contextual impact yields a perceived collision in the full-overlap event even after the moment of full overlap has already passed. The findings from these studies and from the present study suggest that our perception is not a conservative interpretation of sequential input, but it is a dynamic reconstruction process in which new information modifies the immediate past. The present study should add a new insight that the causal context implied in a subsequent event affects this dynamic reconstruction process.

The novel finding in the present study is that the multiple contexts should competitively affect the reconstruction of the temporal structure. The falling ball could affect objects before contacting the floor through causal links interpreted as “magnetic repulsion” or “wind pressure” if subsequent events could fit the interpretations. This view is supported by correlation between PSE shifts and causality rating, and the results where the participants who were not aware of the interpretation about the preceding effect of the falling ball were not affected by the preceding causal context.

The current findings fit well with the Bayesian framework if one assumes the temporal order of events was reconstructed among certain temporal windows (i.e., not too much time lapse). As described earlier, Bayesian inference enables us to model uncertainty about the world by combining prior probabilities with observational, sensory evidence to infer the most probable interpretation of the environment. In the present case, priors would be a complex distribution combining distributions concerning the context of the preceding effect (e.g., magnetic force) and subsequent effects (e.g., impact force), and would competitively interact (Jepson et al., 1996; Yuille and Bülthoff, 1996; Wu et al., 2009). A feature (i.e., type of movement) contained in the subsequent event would activate particular priors to reconstruct a temporal structure. The Type A movement would not activate the priors which consist of preceding causality, or would activate the priors with a small probability, because this type of movement does not fit to any of preceding effects. While for the Type B or C movements, the priors concerning preceding causality have a greater probability, and the mixture of priors concerning each of the preceding and subsequent causalities are competitively activated. Consequently, the shifting of the PSE became relatively small compared to that observed with reference to Type A stimuli. In contrast, Type D stimuli did not affect perceived timing. This would be because knowledge about the causal link between ball contact and color change would be less rigid, and the distribution of duration between two events is more broad (i.e., it is frequent that there is a delay between switch and light, and the lengths of the delay are not constant) than those of ball movements.

In the analysis, I showed that participants who could not report about the preceding effect were not only affected by the preceding context, but they were also less affected by subsequent contexts. These observers would be formulated as observers who are relying more on observation (likelihood) than on prior probabilities in the Bayesian framework. For such observers, prior probabilities concerning the preceding context became relatively small and, in such a case, the context would not be activated. Thus, the context did not affect the temporal perception and the corresponding causal context might therefore not be perceived.

Although I stressed that prediction is not necessary to observe a temporal modulation (causal binding), this does not mean that I intend to exclude the contribution of prediction from the causal binding. As expressed in the Introduction section, the role of prediction also can be formulated in the Bayesian framework. Whichever causal contexts are given in advance or are given in the stimulus, they are used by the brain to decrease the ambiguity in observation, and, consequently, temporal perception is modulated so as to accord with the causal context. In this framework, intention could be thought of as one example of predictive causal links (Buehner, 2012). Interestingly, the present study predicts that there might be a case in which causality prolongs a temporal interval of two events if a causal context is appropriately provided. In further study, investigations to reveal how causal contexts given in a predictive and retrospective manner interact with each other, or demonstrations which show prolongation of temporal intervals of two events are expected.

In this framework, not only perceived temporal order but also the causal structure itself automatically should be determined from the combination of priors and observational input. This view corresponds to the supposition that the causal structure of the world can be directly perceived (Michotte, 1963; Schlottmann and Shanks, 1992; Rolfs et al., 2013). In other words, the detection of causality should be processed in an earlier stage, though the causality has been thought of as higher-level properties. Here, I speculate that linguistic expressions about causal contexts might involve a different process from the detection of causality, and be processed after the perception of causal structures. On this point, previous research indicated that the causal perception and causal inference should be dissociating processes (Schlottmann and Shanks, 1992; Roser et al., 2005). These different processing strategies should lead to various types of introspections in the interviews, and might sometimes lead to a discrepancy between perception and expression. Therefore, I do not think that participants who did not report the preceding effect and participants who were unsusceptible to the preceding effect completely correspond, but they should be correlated. Future study using another subgrouping, such as that based on brain activity, would be expected to divide the participants to allow for the investigation of individual differences in causality perception.

There remains a question of why it had been difficult to observe temporal modulation related to causality with the classic ball-collision stimulus or some other stimuli until Bechlivanidis and Lagnado (2016) and this study examined them. Here, we can identify several differences between previous stimuli and the current stimuli: Contribution of gravity, nature of the animation, three-dimensional setting, and the existence of the floor. Here, I will particularly discuss the existence of the floor, which could be a conductor for the energy of the ball's collision in some displays. This setting prevented participants from observing the direct collision of the ball and objects; the observation of direct collision should increase the reliability of the sensory evidence in a Bayesian formulation. It is known that ambiguity of sensory evidence increases the contribution of prior knowledge. Reconstructed temporal structures could be separated from structures supported by sensory input. This is also the case in the stimuli used in Bechlivanidis and Lagnado's (2016) study; there was an object which can be considered to mediate the energy, and there was a gap between this object (cause) and rightmost object (effect). In their study, there was 350 ms delay between the start of the rightmost object and that of the middle object in their stimulus, which is larger than the temporal shifts obtained in the present study. One reason for the discrepancy might result from the difference in the number of trials conducted by each participant. In Bechlivanidis and Lagnado's (2016) study, the experiment was conducted using a between-subjects design, and the participants observed the causal display only a few times. On the other hand, participants in the present study repeatedly observed causal displays, which might have increased their sensitivity to the temporal structure of the stimuli and increased their accuracy in the task.

Taken as whole, the present study demonstrated that the perceived temporal orders of the cause and effect were transposed according to the perceived causality. Moreover, the results of experiments indicated the competitive effects of multiple causalities on temporal perception, and these results are well-suited to the explanation based on the Bayesian estimation. However, there remain many questions about how and where causality (and its mediating force) are represented and processed in the brain, or what kinds of causal contexts are stored and used by the brain. It is expected that the stimuli introduced in the present study would be helpful for future studies to explore these questions.

## Author contributions

The author confirms being the sole contributor of this work and approved it for publication.

## Funding

This study was supported by JSPS KAKENHI Grant Number 24500335.

### Conflict of interest statement

The author declares that the research was conducted in the absence of any commercial or financial relationships that could be construed as a potential conflict of interest.
